# Establishing Normative Data for the Number Cancelation Test Among Children in Kindergartens and Primary Schools in China

**DOI:** 10.3389/fpsyt.2022.788825

**Published:** 2022-04-11

**Authors:** Yachun Xie, Hongan Wang, Yuxin Chen, Fulin Liu, Mengmeng Yao, Lei Zhang, Panting Liu, Qin Hong, Xia Chi, Dongchuan Yu

**Affiliations:** ^1^Women’s Hospital of Nanjing Medical University (Nanjing Maternity and Child Health Care Hospital), Nanjing, China; ^2^Key Laboratory of Child Development and Learning Science of Ministry of Education, School of Biological Science and Medical Engineering, Southeast University, Nanjing, China; ^3^Henan Provincial Medical Key Lab of Child Developmental Behavior and Learning, The Third Affiliated Hospital of Zhengzhou University, Zhengzhou, China; ^4^Henan Provincial Medical Key Lab of Language Rehabilitation for Children, Sanmenxia Center Hospital, Sanmenxia, China

**Keywords:** Number Cancellation Test, attention, visual-motor integration, learning disabilities, attention deficit/hyperactivity disorder

## Abstract

This study aimed to suggest an attention assessment tool using a Digital Pen for measuring the temporal-spatial parameters during the Number Cancelation Test (NCT), and then to establish the normative data for the NCT among children in kindergartens and primary schools in China by recruiting a total of 989 children (496 males). Four measures, i.e., selective attention (SA), speed of cognitive processing (SpC), averaged time of circlings (ATC), and averaged circumference of circled curves (ACCC), were proposed to evaluate the NCT performance. They basically have a development trend with fast speed in the beginning before Grade 1 or 2 of primary schools, and then enter an extremely slow development period (with ceiling or floor effect). SA and SpC have gender and grade main effects, while ATC and ACCC have the grade main effect, only. In particular, females have higher SA scores than males in middle class of kindergarten, and Grade 2–Grade 5 of primary school, but no gender differences in other grades; females have higher SpC scores than males in middle class of kindergarten, and Grade 3–4 of primary school, but no gender differences in other grades. More importantly, in clinical practice, if SA or SpC measure of a child is below than the 5th centile (i.e., p5 level) of his/her grade-specific normative data, then this child may be predicted to have a high-risk of learning disabilities. Findings suggest that the proposed method can be used for early screening of learning disabilities by setting appropriate cut-off values.

## Introduction

Attention is the basic of complex functions involving several different cognitive and emotional processes and abilities (1, 2). Although there are several definitions and subdivisions of attention capacity, selectivity is arguably the central defining quality of attention and is largely dependent on the frontal lobe (1, 2). The presence of attentional deficits may have a long-lasting impact on daily learning and living. Hence, it is significant to develop precise and accurate attention assessment tools for early detection of attentional deficits (1, 2).

Cancelation tests (3–16) are widely used for measuring individuals’ ability to simultaneously search and scan all stimuli of a certain type (targets) while ignoring stimuli of all other types (distractors). Their clinical utility has been reported in the evaluation of visual attention, associated with a wide range of neurological and psychiatric disorders, such as attention deficit/hyperactivity disorder (ADHD) (3, 4), learning disabilities (LDs) (5), visuospatial neglect syndrome (6, 7), stroke (8), Alzheimer’s disease (9), mild cognitive impairment (10), Parkinson’s disease (11), epilepsy (12), depression (13), and traumatic brain injury (14). Thus far, previous results have basically focused on adults, but only a few studies have been conducted specifically to children (7).

On the other hand, dependent on the traditional paper-and-pencil test, the cancelation tests may have some disadvantages and limitations in performance measures and scoring processes. First, typical performance measures of the cancelation tests include the number of omissions, the number of correct responses, the total number of cancelations, and completion time, but cannot consider the temporal-spatial features from the perspective of handwriting kinematics, such as pre-movement time (initiating), movement time (moving pen to a stimulus), drawing time (completing a cancelation), circumference of a drawn curve, real-time spatial positions (trajectory) of drawing, and the time sequence of drawings. Second, the manual counting method is utilized in the scoring processes of the cancelation tests and thereby, is inconvenient in clinical applications.

Taken together, the current study aimed to suggest an attention assessment tool using a Digital Pen (with an embedded smart mini-camera) for measuring the temporal-spatial features during the Number Cancelation Test (NCT) (15, 16). According to China’s school system, kindergartens are divided into three grades, and primary schools are divided into six grades. A total of 989 children in kindergartens and primary schools were recruited to establish normative data for the NCT among children in kindergartens and primary schools in China. To our knowledge, this is the first time to report normative data (e.g., percentiles for each grade group) of the NTC for such a wide range of children (especially for Chinese children). By setting appropriate cut-off values for these normative data of the NTC, the suggested method had the potential capability for early screening of LDs. This study also investigated if and how the gender and grade influence these temporal-spatial features during the NCT. The internal consistency, test-retest reliability, and validity of the suggested method were discussed as well.

## Materials and Methods

### Participants

The current study was conducted in Nanjing, Jiangsu Province, China, between September 2020 and March 2021, and selected participants with a multistage stratified random sampling. According to the districts’ rankings of GDP per person in 2019, the districts of Nanjing were divided into three levels, i.e., Strong (>130,000 RMB), Medium (100,000–130,000 RMB), Weak (<100,000 RMB). By a random-number generator *via* Matlab Statistic Toolbox (R2012b), we conducted a sequence of random operations as follows. First, we selected randomly three districts (corresponding to Strong, Medium, and Weak level, respectively), and two primary schools and one kindergarten for each district. Then, we chose randomly one class from a grade of each primary school, and three classes from a grade of each kindergarten. Furthermore, we selected randomly ten males and ten females from a class of each primary school or kindergarten. By steps above, we recruited a total of 720 children from primary schools and 540 children from kindergartens. We excluded 38 children with a history of previous neurological or psychiatric disorders, or children who had repeated a grade. We further excluded 233 children, who cannot submit their experimental data due to loss of data or refusal to attend the experiments. Hence, 989 children (496 males) were finally considered in the current study.

All study procedures and research methods were carried out in accordance with the Declaration of Helsinki (17) by the World Medical Association concerning human experimentation, and were approved by the Research Ethics Committee at Southeast University. Informed consent was obtained from all parents of participating children and oral consent was obtained from all participating children. Each child received an age-appropriate toy after completing the study.

### Procedure

We suggested a newly developed tool using a Digital Pen for recording the temporal-spatial features during the NCT, and obtained the NCT measures for attention assessment. More importantly, we aimed to establish the normative data for the NCT measures from 989 children in kindergartens and primary schools. In addition, it was hypothesized that by setting appropriate cut-off values for these normative data, the NCT measures can be used for early screening of LDs. To test this hypothesis, we suggested using the Pupil Rating Scale Revised (PRS) questionnaire (18–21), which has been widely used for the screening of LDs and is assumed to be a golden standard of LDs in this study, to investigate the validity of screening using the NCT measures.

Participating children were instructed to complete the NCT task. The teachers of participants from primary schools were required to complete the PRS questionnaire. The participants took the tests at school in a quiet room.

### Number Cancelation Test Task

The examiner sat in front of a participant and presented the participant with a standard B5-size paper showing a series of numbers arranged in organized arrays with 26 rows and 40 columns, and the participant hold the Digital Pen with an embedded smart mini-camera correctly. The test instruction given to participants was that “Honey, there are many numbers below. You should find the number “3” (the targeted number) and draw a circle on it, but ignore all other numbers (distractors), as quickly as possible within 2 min.” A laptop linked to the Digital Pen with Bluetooth wireless technology, and recorded the data generated and transferred from the Digital Pen. The technical advantage of the Digital Pen was the usage of a smart mini-camera (being embedded in the Digital Pen), designed to measure the temporal-spatial parameters during the NCT. In the current study, four parameters were suggested as the scores to measure individuals’ performance during the NCT, including:

(1) Speed of cognitive processing (SpC) was defined as:


(1) $$S\,p\,C=M\,\sum_{i=1}^{N}R_{i}$$

where *M* was the amount of numbers in one row (here *M* = 40); *N* was the total number of rows to be circled; *R*_*i*_=1 represented the case if any number in the *i*-th row has been circled; and *R*_*i*_=0 represented the case if no number in the *i*-th row has been circled.

(2) Selective Attention (SA) was defined as:


(2) $$S\,A=\frac{1}{T}\,\frac{m-\omega }{m+o}\times \text{SpC}$$

where *o* was the amount of omitted targets; ω was the number of distractors being circled; and *m* was the total amount of targets that should be circled; *T* was the task time (here *T* = 120); SpC was defined by Eq. 1.

(3) Averaged time of circlings (ATC) was defined as:


(3) $$A\,T\,C=\frac{1}{n}\times \sum_{i=1}^{n}t_{i}$$

where *n* was the amount of numbers being circled; and *t_i* was the time to circle the *i*-th number.

(4) Averaged circumference of circled curves (ACCC) was defined as:


(4) $$A\,C\,C\,C=\frac{1}{n}\times \sum_{i=1}^{n}C_{i}$$

where *n* was the amount of numbers being circled; and *C_i* was the circumference of the curve circling the *i*-th number.

**FIGURE 1:**
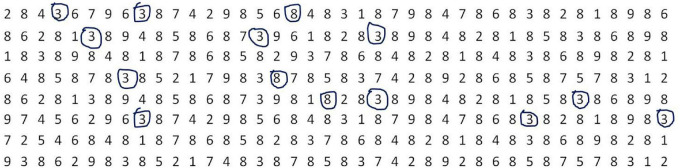
An example illustrated a case that a participant completed only 6 rows in 2 min, where “3” is the targeted number and all other numbers are distractors. In this example, 14 numbers were circled, where the amount number of “3” was 11 and that of “8” was 3. In addition, the amount number of omitted “3” was 12, where the number of omitted “3” in the 1st, 2nd, 3rd, 4th, 5th, and 6th row was 2, 1, 3, 3, 2, and 1, respectively.

An example (see Figure 1) illustrated a case that a participant completed only 6 rows in 2 min, where “3” was the targeted number and all other numbers were distractors. It is easy to see from Figure 1 that the whole task lasted 2 min (i.e., *T* = 120); the total amount of numbers being circled was 14 (i.e., *n* = 14); the number of omitted targets was 12 (i.e., *o* = 12), where the amount of omitted targets in the 1st, 2nd, 3rd, 4th, 5th, and 6th row was 2, 1, 3, 3, 2, and 1, respectively; three distractors (i.e., “8” in this case showing in Figure 1) in the 1st, 4th, and 5th row were circled (i.e., ω=3); the total number of targets that should be circled was 22 (i.e., *m* = 22); the amount of numbers in one row was 40 (i.e., *M* = 40); *R*_1_=*R*_2_=*R*_4_=*R*_5_=*R*_6_=1 because there were at least one number (being circled) in the 1st, 2nd, 4th, 5th, and 6th row; and *R*_3_=0 because no numbers were circled in the 3rd row. Therefore, *SpC* = 40*(*R*_1_ + *R*_2_ + *R*_4_ + *R*_5_ + *R*_6_) = 200; $S\,A=\frac{1}{120}\,\frac{22-3}{22+1}\times 200=0.43$. The parameter ATC can be easily calculated by averaging the time of circling each number. While, the parameter ACCC can be simply calculated by averaging the curve circumference of each circling.

### Pupil Rating Scale Revised Questionnaire

The PRS questionnaire (18–21) has been widely used for the screening of LDs and is assumed to be a golden standard of LDs in this study. It consists of 24 items rated on a 5-point Likert scale, and is comprised of five subscales (including auditory comprehension and memory, spoken language, orientation, motor coordination, and personal-social behavior). The score of each item ranges from 1 (“the lowest”) to 5 (“the highest”). Hence, the total score of the PRS questionnaire ranges from 24 (minimum) to 120 (maximum). Higher score means better learning ability.

The PRS questionnaire includes verbal and non-verbal measures, where verbal and non-verbal measures contain the items from two subscales (i.e., spoken language, and auditory comprehension and memory) and from other three subscales (i.e., orientation, motor coordination, and personal-social behavior), respectively. Therefore, the total score of the verbal measure ranges from 9 (minimum) to 45 (maximum); while the total score of the non-verbal measure ranges from 15 (minimum) to 75 (maximum). It has been verified (18–21) that LDs can be screened by the rule that the participants with verbal measure below than 20, with non-verbal measure below than 40, and with total score below than 65 were suspected to be verbal LD, non-verbal LD, and general LD, respectively. Findings of a large sample research (*n* = 3991) verified (20) that the PRS questionnaire had a high reliability for Chinese children (its reliability coefficients being higher than 0.84 for all subscales).

The teachers of participants from primary schools were instructed to complete the PRS questionnaire and evaluate participants’ risk of LDs.

### Centile Curves

Centile curves of the NCT measures were computed using the LMS method (22–24), which obtains normalized growth centile standards by optimizing three curves representing the skewness (L), median (M), and coefficient of variation (S). The resulting L, M, and S curves contain the information to draw any centile by the following formula (22–24):


(5) $$C_{100\,\alpha }\,\left(t\right)=M\,\left(t\right)\,{\left[1+L\,\left(t\right)\,S\,\left(t\right)\,Z_{\alpha }\right]}^{1/L\,\left(t\right)}$$

where *Z*_α_ is the normal equivalent deviate of size α. For participants, the 5th, 10th, 15th, 25th, 50th, 75th, and 90th centiles were chosen as age-specific reference values. Centile curves, shown in Eq. 5, were calculated with R language (version 4.0.2).

### Cut-Off Values for Screening of Learning Disabilities

Participants were suspected to be LD if their PRS measures meet one of the following conditions: (i) The score of verbal measure is below than 20; (ii) The score of non-verbal measure is below than 40; (iii) The total score is below than 65. It is hypothesized that a participant is suspected to be LD, if the following condition can be satisfied


(6) $$N\,C\,T^{\left(j\right)}<\beta_{p\,i}^{\left(j\right)}$$

where *NCT*^(*j*)^ is the *j*-th measure of NCT; $\beta_{p\,i}^{\left(j\right)}$ is the cut-off value of *NCT*^(*j*)^ with the *pi*-th centile.

The screening of LD by PRS measures is assumed to be a golden standard in this study. By this way, one can investigate the screening performance based on the NCT measures. In particular, this study revealed that how the screening accuracy of LD is influenced by the cut-off values $\beta {}_{p\,i}^{\left(j\right)}$.

### Statistical Analysis

**FIGURE 2:**
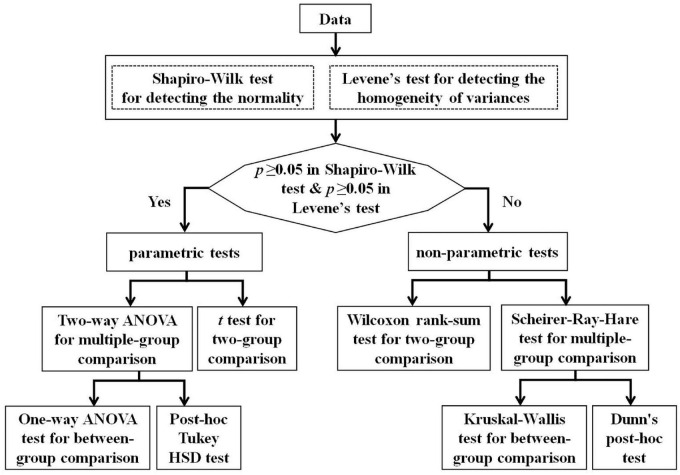
The flowchart of statistical analysis.

We aimed to investigate how the gender and grade influence the measures (i.e., SpC, SA, ATC, ACCC) quantifying the NCT performance of participants. Hence, we conducted a series of two-way ANOVA for these measures, according to the flowchart (see Figure 2). We verified that our data (i.e., the NCT measures) failed to pass both normality test and variance homogeneity test, so we conducted a series of non-parametric two-way ANOVA procedures (i.e., Scheirer–Ray–Hare tests) for the NCT measures. In addition, we used the Kruskal Wallis method and Dunn’s *post-hoc* test for multiple comparisons with Benjamini-Hochberg procedure to control the false discovery rate. All statistical analysis above was conducted with R language (version 4.0.2).

## Results

### General Information of Participants

The current study actually investigated a total of 989 children, including 496 males and 493 females. The ratio of males to females was 1.006:1 and the participants were distributed in 9 grade groups, ranging from GR1 (corresponding to junior class of kindergartens) to GR9 (corresponding to Grade 6 of primary schools), see Table 1 for detailed information. It has verified that there was no significant gender difference (_χ^2_ = 10.43, *p* = 0.24).

**TABLE 1:** Demographic characteristics of participants.

Grade groups	Males (*N*, %)	Total (*N*)	Age (years)
GR1 (Junior Class of kindergarten)	73 (49.7)	147	3.91 ± 0.27
GR2 (Middle Class of kindergarten)	84 (45.65)	184	4.85 ± 0.27
GR3 (Senior Class of kindergarten)	89 (53.61)	166	5.91 ± 0.29
GR4 (Grade 1 of primary school)	59 (61.46)	96	6.84 ± 0.31
GR5 (Grade 2 of primary school)	49 (49.49)	99	7.84 ± 0.30
GR6 (Grade 3 of primary school)	49 (50)	98	8.76 ± 0.28
GR7 (Grade 4 of primary school)	42 (53.16)	79	9.82 ± 0.34
GR8 (Grade 5 of primary school)	34 (43.59)	78	10.80 ± 0.30
GR9 (Grade 6 of primary school)	17 (40.48)	42	11.80 ± 0.27
Total	496 (50.15)	989	N/A

### Main Effects of Age and Gender

In this study, we aimed to investigate how the gender and grade (age) influence the four parameters (i.e., SpC, SA, ATC, ACCC). According to the statistical flowchart shown in Figure 2, we verified that our data (i.e., the NCT measures) failed to pass both normality test and variance homogeneity test (*p*’s ≥ 0.05). Hence, we conducted a series of non-parametric two-way ANOVA procedures (i.e., Scheirer–Ray–Hare tests) to reveal the gender and grade main effects as well as for their interaction. Our findings showed that: (i) The main effect of grade is significant for ATC and ACCC (Gender: *p*’s > 0.05; Grade: *p*’s < 1 × 10^–4^; Gender * Grade: *p*’s > 0.05); and (ii) The main effects of gender and grade were significant (but there was no interaction effect) for SpC and SA (Gender: *p*’s < 0.01; Grade: *p*’s < 1 × 10^–4^; Gender * Grade: *p*’s > 0.05).

**FIGURE 3:**
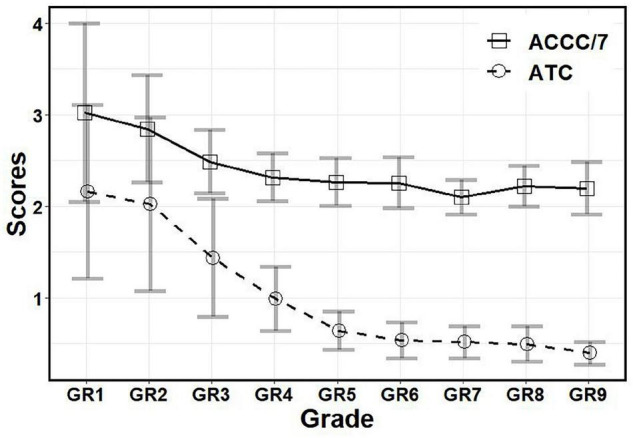
The scores of ATC and ACCC in different grades. ATC, Averaged time of circlings; ACCC, Averaged circumference of circled curves; GR1, Junior Class of kindergarten; GR2, Middle Class of kindergarten; GR3, Senior Class of kindergarten; GR4, Grade 1 of primary school; GR5, Grade 2 of primary school; GR6, Grade 3 of primary school; GR7, Grade 4 of primary school; GR8, Grade 5 of primary school; GR9, Grade 6 of primary school.

**FIGURE 4:**
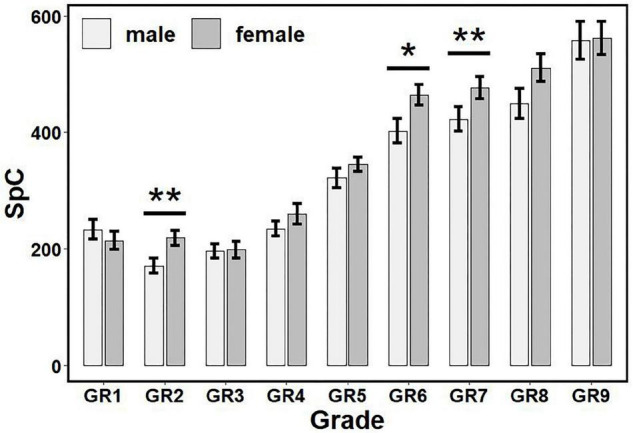
The influence of gender and grade on SpC scores. There are gender differences in GR2, GR6, and GR7. * *p* < 0.05, ** *p* < 0.01. SpC, speed of cognitive processing; GR1, Junior Class of kindergarten; GR2, Middle Class of kindergarten; GR3, Senior Class of kindergarten; GR4, Grade 1 of primary school; GR5, Grade 2 of primary school; GR6, Grade 3 of primary school; GR7, Grade 4 of primary school; GR8, Grade 5 of primary school; GR9, Grade 6 of primary school.

**FIGURE 5:**
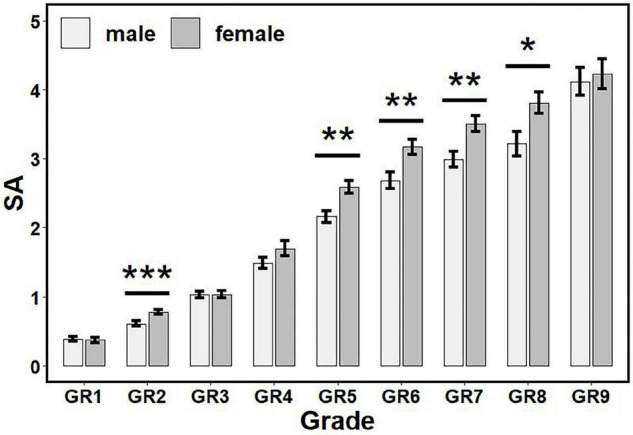
The influence of gender and grade on SA scores. There are gender differences in GR2, GR5–GR8. * *p* < 0.05, ** *p* < 0.01, *** *p* < 0.001. SA, Selective Attention; GR1, Junior Class of kindergarten; GR2, Middle Class of kindergarten; GR3, Senior Class of kindergarten; GR4, Grade 1 of primary school; GR5, Grade 2 of primary school; GR6, Grade 3 of primary school; GR7, Grade 4 of primary school; GR8, Grade 5 of primary school; GR9, Grade 6 of primary school.

According to the statistical flowchart shown in Figure 2, we further utilized the Kruskal Wallis method and Dunn’s *post hoc* for multiple comparisons with Benjamini-Hochberg procedure to control the false discovery rate. Figures 3–5 summarized our results and verified that:

(i) For ATC (see Figure 3): Children in GR1-GR4 had higher scores than other grades (*p*’s < 0.05, adjusted), but there was no significant difference between children in GR1 and GR2 (*p* > 0.05, adjusted); there were no significant differences among GR7-GR12 (corresponding to Grades 3–6) (*p*’s > 0.05, adjusted).
(ii) For ACCC (see Figure 3): Children in GR1-GR3 had higher scores than other grades (*p*’s < 0.05, adjusted), but there was no significant difference between children in GR1 and GR2 (*p* > 0.05, adjusted); there were no significant differences between children in Grades 1–3 and between Grades 2–6 (*p*’s > 0.05, adjusted).
(iii) For SpC (see Figure 4): Females had higher scores than males in GR2, GR6 and GR7 (*p*’s < 0.05), but no significant differences had been found in other grades (*p*’s > 0.05).
(iv) For SA (see Figure 5): Females had higher scores than males in GR2, GR5-GR8 (*p*’s < 0.05), but no significant differences had been found in other grades (*p*’s > 0.05).

### Internal Consistency and Test-Retest Reliability

The internal consistency of these four temporal-spatial indexes was detected by the linear correlation analysis. Table 2 summarized our results and showed that there were high correlation coefficiencies (thus high internal consistency) among these four indexes. In addition, we also verified that the consistency among experimenters was 0.997, implying that the operation of all experimenters was highly consistent.

**TABLE 2:** Internal consistency of the NCT.

	SpC	SA	ATC	ACCC
SpC	–			
SA	0.81↑	–		
ATC	–0.53↑	-0.68↑	–	
ACCC	–0.25↑	–0.43↑	0.61↑	–

*↑ p < 0.0001.*

*NCT, Number Cancelation Test; SpC, Speed of cognitive processing; SA, Selective Attention; ATC, Averaged time of circlings; ACCC, Averaged circumference of circled curves.*

To assess the test-retest reliability by calculating intraclass correlation coefficients (ICCs) for all parameters, 117 participants were asked to undergo a second NCT test, where time between two assessments was 14 days. It was very encouraging that ICCs of SA, ATC, SpC, and ACCC are 0.81, 0.81, 0.655, and 0.62, respectively. This implies that according to Fleiss’s rule, the test-retest reliability of SA and ATC are excellent (ICCs > 0.8), while the test-retest reliability of SpC and ACCC are good (ICCs > 0.6).

To summarize, the internal consistency, the consistency among experimenters, and the test-retest reliability are acceptable.

### Test Validity

The PRS questionnaire has been widely used in evaluating the risk of learning disabilities in children. Findings revealed (see Table 3) that SpC, SA and ACCC scores were correlative with the indexes of PRS questionnaire (*p*’s < 0.05).

**TABLE 3:** Correlation between the NCT indexes and the PRS scores.

	SpC	SA	ATC	ACCC
Auditory comprehension and memory	0.08*	0.14**	-0.006	-0.11*
Spoken language	0.10*	0.16***	-0.01	-0.10*
Orientation	0.11*	0.17***	-0.019	-0.11*
Motor coordination	0.14**	0.19***	-0.006	-0.13**
Personal-social behavior	0.11*	0.19***	-0.056	-0.15**
Verbal measure	0.09*	0.17***	-0.0097	-0.11*
Non-verbal measure	0.12*	0.20***	-0.05	-0.14**
Total score	0.12*	0.19***	-0.035	-0.13**

*p < 0.05, ** p < 0.01, ***p < 0.001.

*NCT, Number Cancelation Test; PRS, Pupil Rating Scale Revised; LD, learning disability; SpC, Speed of cognitive processing; SA, Selective Attention; ATC, Averaged time of circlings; ACCC, Averaged circumference of circled curves.*

### Normative Data

The normative data is naively a simple reference range. Clearly, in our case that the measures are strongly dependent on grade, the reference ranges change with grade and lead to inconvenience in practice. Fortunately, centile curves (22–24) overcome the drawback of the grade-related reference ranges, and provide a visualizing outline to present the normative data. Centile curves were calculated according to Eq. 5 for SA, ATC, ACCC, and SpC measures. Any percentile can be evaluated for each grade group by the formula shown in Eq. 5. In this study, the 5th, 10th, 15th, 25th, 50th, 75th, and 90th centiles were chosen as grade-specific reference values. Tables 4–7 summarizes our results, and showed the 5th, 10th, 15th, 25th, 50th, 75th, and 90th centiles for SA, ATC, ACCC, and SpC measures for each grade group.

**TABLE 4:** The 5th, 10th, 15th, 25th, 50th, 75th, and 90th centiles for Selective Attention (SA) measure for different grade groups.

Grade groups	P5	P10	P15	P25	P50	P75	P90
GR1 (Junior Class of kindergarten)	0.02	0.06	0.08	0.14	0.30	0.53	0.74
GR2 (Middle Class of kindergarten)	0.11	0.25	0.33	0.46	0.67	0.90	1.16
GR3 (Senior Class of kindergarten)	0.33	0.46	0.58	0.79	0.97	1.31	1.60
GR4 (Grade 1 of primary school)	0.51	0.79	1.02	1.24	1.55	2.14	2.43
GR5 (Grade 2 of primary school)	1.50	1.62	1.69	2.01	2.51	2.94	3.30
GR6 (Grade 3 of primary school)	1.71	2.21	2.33	2.63	2.95	3.48	4.13
GR7 (Grade 4 of primary school)	2.12	2.25	2.38	2.59	3.28	3.80	4.16
GR8 (Grade 5 of primary school)	2.07	2.24	2.37	2.69	3.40	4.25	5.07
GR9 (Grade 6 of primary school)	2.59	2.85	3.09	3.66	4.17	4.83	5.22

**TABLE 5:** The 5th, 10th, 15th, 25th, 50th, 75th, and 90th centiles for Speed of cognitive processing (SpC) measure for different grade groups.

Grade groups	P5	P10	P15	P25	P50	P75	P90
GR1 (Junior Class of kindergarten)	80	80	80	120	200	280	440
GR2 (Middle Class of kindergarten)	40	80	80	120	160	240	360
GR3 (Senior Class of kindergarten)	80	80	120	120	160	240	360
GR4 (Grade 1 of primary school)	120	120	160	200	240	320	372
GR5 (Grade 2 of primary school)	200	240	240	280	360	400	480
GR6 (Grade 3 of primary school)	280	320	330	360	440	480	640
GR7 (Grade 4 of primary school)	280	320	360	360	440	510	560
GR8 (Grade 5 of primary school)	280	304	320	360	480	560	720
GR9 (Grade 6 of primary school)	346	360	400	480	560	640	708

**TABLE 6:** The 5th, 10th, 15th, 25th, 50th, 75th, and 90th centiles for Averaged time of circlings (ATC) measure for different grade groups.

Grade groups	P5	P10	P15	P25	P50	P75	P90
GR1 (Junior Class of kindergarten)	0.77	1.04	1.23	1.52	2.00	2.85	3.43
GR2 (Middle Class of kindergarten)	0.94	1.06	1.18	1.42	1.81	2.39	3.27
GR3 (Senior Class of kindergarten)	0.65	0.85	0.92	1.03	1.30	1.80	2.09
GR4 (Grade 1 of primary school)	0.48	0.49	0.60	0.68	0.90	1.17	1.41
GR5 (Grade 2 of primary school)	0.36	0.38	0.41	0.47	0.57	0.68	0.83
GR6 (Grade 3 of primary school)	0.32	0.35	0.36	0.37	0.46	0.57	0.70
GR7 (Grade 4 of primary school)	0.28	0.33	0.35	0.38	0.50	0.62	0.74
GR8 (Grade 5 of primary school)	0.27	0.29	0.32	0.37	0.45	0.59	0.78
GR9 (Grade 6 of primary school)	0.22	0.27	0.28	0.32	0.38	0.43	0.55

**TABLE 7:** The 5th, 10th, 15th, 25th, 50th, 75th, and 90th centiles for Averaged circumference of circled curves (ACCC) measure for different grade groups.

Grade groups	P5	P10	P15	P25	P50	P75	P90
GR1 (Junior Class of kindergarten)	7.70	14.19	15.59	17.20	20.98	24.72	29.65
GR2 (Middle Class of kindergarten)	14.19	15.80	16.83	17.74	19.21	21.61	24.56
GR3 (Senior Class of kindergarten)	13.34	14.64	15.50	16.13	17.49	18.75	19.99
GR4 (Grade 1 of primary school)	13.39	13.86	14.14	14.91	16.19	17.10	17.16
GR5 (Grade 2 of primary school)	13.05	13.62	13.70	14.30	15.67	17.05	17.94
GR6 (Grade 3 of primary school)	12.74	13.45	14.00	14.45	15.36	16.76	18.02
GR7 (Grade 4 of primary school)	12.36	13.07	13.30	13.99	14.64	15.47	16.09
GR8 (Grade 5 of primary school)	13.32	13.78	13.93	14.55	15.49	16.37	17.42
GR9 (Grade 6 of primary school)	12.87	13.36	13.46	13.89	15.15	16.33	17.33

### Cut-Off Values for Screening of Learning Disabilities

It was interesting to detect whether these measures (i.e., SpC, SA, ATC, and ACCC) can be used to screen LDs, according to Eq. 6. Tables 8, 9 summarized our results and showed that SA and SpC measures could be used to screen LDs with high accuracy (bigger than 0.73) by setting appropriate cut-off values, but ATC and ACCC measures were inconsistent for all grades and failed to screen LDs (not shown). In addition, we suggest from Tables 8, 9 that the 5th centile (i.e., p5 level) can be considered as the optimal cut-off value of SA and SpC measures because the screening accuracy reaches its highest accuracy for all grade groups. This implies that if SA or SpC measure of a child is below than the 5th centile (i.e., p5 level) of his/her grade-specific normative data, then this child may be predicted to have a high-risk of LDs.

**TABLE 8:** Screening accuracy of learning disabilities (LDs) based on Selective Attention (SA) measure by setting cut-off values ranging from 5th (p5) to 90th (p90) centiles for different grade groups.

Grade groups	P5	P10	P15	P25	P50	P75	P90
GR4 (Grade 1 of primary school)	0.85	0.84	0.82	0.76	0.53	0.37	0.26
GR5 (Grade 2 of primary school)	0.85	0.83	0.81	0.75	0.49	0.33	0.20
GR6 (Grade 3 of primary school)	0.82	0.79	0.79	0.73	0.51	0.35	0.23
GR7 (Grade 4 of primary school)	0.73	0.71	0.73	0.71	0.64	0.42	0.35
GR8 (Grade 5 of primary school)	0.78	0.78	0.73	0.68	0.53	0.42	0.30
GR9 (Grade 6 of primary school)	0.87	0.85	0.81	0.72	0.54	0.33	0.19

*It is clear that the 5th centiles (p5) obtain the highest accuracy for all grade groups.*

**TABLE 9:** Screening accuracy of learning disabilities (LDs) based on Speed of cognitive processing (SpC) measure by setting cut-off values ranging from 5th (p5) to 90th (p90) centiles for different grade groups.

Grade groups	P5	P10	P15	P25	P50	P75	P90
GR4 (Grade 1 of primary school)	0.87	0.87	0.78	0.56	0.44	0.31	0.23
GR5 (Grade 2 of primary school)	0.82	0.81	0.81	0.67	0.33	0.24	0.11
GR6 (Grade 3 of primary school)	0.81	0.77	0.77	0.64	0.38	0.30	0.14
GR7 (Grade 4 of primary school)	0.74	0.73	0.67	0.67	0.55	0.45	0.36
GR8 (Grade 5 of primary school)	0.78	0.78	0.71	0.67	0.53	0.40	0.27
GR9 (Grade 6 of primary school)	0.83	0.83	0.78	0.63	0.52	0.24	0.22

*It is clear that the 5th centiles (p5) obtain the highest accuracy for all grade groups.*

## Discussion

This study aimed to suggest an attention assessment tool using a Digital Pen with an embedded smart mini-camera for recording the temporal-spatial features during the NCT. The advantages of the suggested method are twofold. First, it considers not only the traditional static features (e.g., the number of omissions, the number of correct responses, the total number of cancelations), but also the dynamic features, such as drawing time (completing a cancelation), circumference of circled curves, drawing speed, real-time spatial trajectory of drawings, and the time sequence of drawings. Second, the suggested method has an automated scoring process, thus provides a more sensitive and accurate measure of process and outcome of attention, motor, and visuospatial performance than traditional administration.

A total of 989 children (496 males) in kindergartens and primary schools were recruited to establish the normative data for the NCT among children in kindergartens and primary schools. Tables 4–7 showed the 5th, 10th, 15th, 25th, 50th, 75th, and 90th centiles of the NCT measures for each grade group. Remarkably, SA, ATC, ACCC, and SpC measures basically have a developmental trend (i.e., increased or decreased continuously with grade), especially after GR2 (Middle Class of kindergarten). To our knowledge, this is the first time to report normative data of the NTC for such a wide range of children (especially in China).

It was verified (see Tables 8, 9) that by setting appropriate cut-off values (e.g., the 5th centile), SA and SpC measures can be used for early screening of LDs with high accuracy (bigger than 0.7). This is consistent with the fact that the prevalence of LDs, as reported by the DSM-5 [APA (25)], is between 5 and 15% in the school population.

The findings showed that SpC and SA measures have gender and grade main effects but no interaction effect, while ATC and ACCC measures have the grade main effect, only. In addition, it was very encouraging (see Figures 3–5) that all measures have a development trend with fast speed in the beginning before Grade 1 or 2 of primary schools, and then enter an extremely slow development period (with ceiling or floor effect). Remarkably, females have higher SpC scores than males in GR2, GR6, and GR7, but no gender differences in other grades; females have higher SA scores than males in GR2, GR5–GR8, but no gender differences in other grades.

It is well established that females have a faster cognitive and social development up to the end of adolescence than males of the same age. Previous research has also shown that gender difference plays a significant role in the evaluation of neurological and psychiatric disorders, and the literature associated with LDs and ADHD supports a higher prevalence in males (26–29). Remarkably, our findings (shown in Figures 3–5) verified that: (i) Females have higher SpC scores than males in GR2, GR6, and GR7, but no gender differences in other grades; and (ii) Females have higher SA scores than males in GR2, GR5–GR8, but no gender differences in other grades. Hence, our findings about the gender difference are consistent with previous studies. However, our results also showed that there were no gender differences of SpC or SA after Grade 5 or 6 of primary schools. These results here might provide some new insights into understanding the gender difference in the evaluation of neurological and psychiatric disorders (26–29). First, it deserves to test whether there are gender differences across the lifespan (especially after Grade 6 of primary schools). Second, the apparent gender differences of LDs or ADHD might be caused by the gender difference of cognitive level at some age period. Third, the diagnostic criteria for LDs or ADHD might be biased or poorly specified for one gender and/or grade group.

Children with LDs may suffer from the deficits of skills in selective and sustained attention, motor inhibition, visuospatial search, planning, organizing, psychomotor speed, intact visual-perception abilities, fine motor coordination, and sensory motor integration. These skills may basically be measured and interpreted by the suggested method measuring temporal-spatial features during the NCT. Hence, it is not surprising that these temporal-spatial features are highly correlative with the scores of the PRS questionnaire. More importantly, it has been revealed that by setting appropriate cut-off values (e.g., the 5th centile), SA and SpC measures can be used for early screening of LDs with high accuracy (bigger than 0.7). In particular, if SA or SpC measure of a child is below than the 5th centile (i.e., p5 level) of his/her grade-specific normative data, then this child may be predicted to have a high-risk of LDs. These findings suggest that our method, in combination with classification using machine learning tools and considering more temporal-spatial features, has the potential for early screening of LDs, and will be investigated in a future research.

## Conclusion

This study aimed to suggest an attention assessment tool measuring temporal-spatial features during the NCT, and then to establish normative data (i.e., percentiles for each grade group) for the NCT among children in kindergartens and primary schools in China. The influence of the gender and grade (age) on the NCT measures have been investigated as well. In clinical practice, if SA or SpC measure of a child is below than the 5th centile (i.e., p5 level) of his/her grade-specific normative data, then this child may be predicted to have a high-risk of learning disabilities. Findings verified that the suggested method has the potential for early screening of LDs by setting appropriate cut-off values, thus allowing for better diagnosis and intervention. Future research is warranted to develop effective personalized programs for remediation and rehabilitation, dependent on an individual’s attention measure scores.

## Data Availability Statement

The raw data supporting the conclusions of this article will be made available by the authors, without undue reservation.

## Ethics Statement

The studies involving human participants were reviewed and approved by Research Ethics Committee at Southeast University. The patients/participants provided their written informed consent to participate in this study.

## Author Contributions

XC and DY developed the idea for the study. YX, YC, FL, MY, LZ, PL, and QH collected the data. YX, YC, HW, XC, and DY did the analyses. DY wrote the manuscript. All authors contributed to the article and approved the submitted version.

## Conflict of Interest

The authors declare that the research was conducted in the absence of any commercial or financial relationships that could be construed as a potential conflict of interest.

## Publisher’s Note

All claims expressed in this article are solely those of the authors and do not necessarily represent those of their affiliated organizations, or those of the publisher, the editors and the reviewers. Any product that may be evaluated in this article, or claim that may be made by its manufacturer, is not guaranteed or endorsed by the publisher.
